# Community-Enhanced Social Prescribing: Integrating Community in Policy and Practice

**DOI:** 10.1007/s42413-020-00080-9

**Published:** 2020-12-02

**Authors:** David Morris, Paul Thomas, Julie Ridley, Martin Webber

**Affiliations:** 1 Centre for Citizenship and Community, University of Central Lancashire, Preston, UK; 2 University of West London, London, UK; 3 International Centre for Mental Health Social Research, Department of Social Policy and Social Work, University of York, York, UK

**Keywords:** Social prescribing, Citizenship, Community, Connected communities, Connecting people, Wellbeing

## Abstract

The NHS Plan is introducing social prescribing link workers into GP surgeries in England. The link workers connect people to non-health resources in the community and voluntary sector, with the aim of meeting individual needs beyond the capacity of the NHS. Social prescribing models focus on enhancing individual wellbeing, guided by the policy of universal personalised care. However, they largely neglect the capacity of communities to meet individual need, particularly in the wake of a decade of austerity. We propose a model of community enhanced social prescribing (CESP) which has the potential to improve both individual and community wellbeing. CESP combines two evidence-informed models – Connected Communities and Connecting People – to address both community capacity and individual need. CESP requires a literacy of community which recognises the importance of communities to individuals and the importance of engaging with, and investing in, communities. When fully implemented the theory of change for CESP is hypothesised to improve both individual and community wellbeing.

## Introduction

Community-enhanced social prescribing (CESP) is a new model of social prescribing combining community engagement, organisational change and individual-level practice which aims to improve both community and individual wellbeing. It provides a way of thinking about the reciprocal value of individual and community wellbeing in the context of primary health care and local communities.

In England there is substantial investment in social prescribing within primary care networks, providing a significant opportunity to improve community wellbeing. The National Health Service (NHS) Long Term Plan (NHS England 2019a) is arguably too narrowly focused on individual outcomes and new social prescribing schemes will need to engage with, and orient themselves more towards, local communities. The CESP model articulated in this paper provides a framework for achieving this.

Key paradigms of community capacity building and connecting individuals to community currently run in parallel rather than being part of an integrated whole. CESP integrates these paradigms into a model which aligns them at theoretical, policy and practice levels. Uniquely, CESP is concerned with community wellbeing as well as individual wellbeing, a necessary component of social prescribing which is largely absent from current policy discourses.

CESP is conceptualised in the context of the UK where health and social care systems are well-developed. The Government-funded NHS provides universal health care, mostly free at the point of delivery. Responsibility for the organisation of health services has been devolved to the four nations (England, Wales, Scotland and Northern Ireland) resulting in some local variation in provision. In Northern Ireland, for example, health and social care services are integrated, whereas in England, Local Authorities have responsibility for the provision of social care. Although CESP specifically relates to the UK context, it is envisaged that it could be applied in other countries where communities are seeking ways to be better connected in order to enhance both community and individual wellbeing.

## UK Policy Context

While the imperative of social prescribing has its contemporary policy roots in the drive to engaging individuals through the provisions of universal personalised care (NHS England 2019c), its long term effectiveness will depend on integrating this with a new formulation of community care and engagement; a take on integration which existing narratives have, to date, largely failed to embrace.

The history of health and social care integration in the UK is a long one. In 2009, Ham and Oldham noted that while the objective of achieving closer integration of health and social care had been crystallised in the Health Act 1999 a decade earlier, as a policy aim it had spanned half a century. Central to this aim has been the desirability of integrating the community services of health and social care sectors. Yet for all the subsequent policy drive of recent years towards its implementation, vehicles for convergence have left communities almost entirely stranded at the roadside. As systems and models for service commissioning and delivery are repeatedly engineered and re-engineered towards meeting the vision for effective cohesion in health and social care organisations, the prize of integrating both of these sectors with that of community sector organisations and communities themselves has barely been considered.

Clearly, integration has long been prominent in health policy as a critical dimension for bringing about a focus on community health. The Five Year Forward View for the NHS cited the necessity for a new community health approach, setting out proposals for multi-specialty community providers and primary care based models to achieve them (NHS England 2014). Such models exemplified a basis for closer partnerships between primary, community, mental health and social care services and were viewed as effective (Collins 2016). These new models of integration have gone on to shape a vision for the NHS in which the transformation needed to ensure progress on integration is to be pursued through strategic partnerships, to be consolidated by 2021, as Integrated Care Systems.

So, although the multiple complexities of integration have made the path to its achievement inordinately long, it seems that something like an endpoint is in sight. But an endpoint at which the success of integration is judged by its scope to overcome the “organisational, professional, legal and regulatory boundaries within the health and social care sectors” (National Audit Office 2017, p. 5) is surely not really an endpoint at all. In defining integration as being about “improving patient outcomes, satisfaction and value for money” (ibid, p.5) while remaining silent on how the communities of which patients are members are to be engaged, is no way to ensure that they are placed “at the centre of the design and delivery of care” (ibid, p.5). Rather, it demonstrates a process of policy formulation in which the dimensions of management and governance in integration are privileged over its human values and social potential.

Meanwhile in social care, the value of communities in shaping care models and their implementation has been an implicit guiding notion for many decades, having at times been explicitly advocated as the necessary orientation for the social work profession. Notably, the Barclay report - commissioned by government from a two-year review of social work in England and Wales - recommended, through processes akin to today’s ‘co-production’, a re-casting of professional practice towards the purposeful engagement of informal carers and communities. For social work, Barclay (1982) recommended an active professional relationship of brokerage and support for individuals’ social networks of support. Critically, alongside restatement of what then was a largely consensual ethic of citizen entitlement to public welfare services, the report envisaged a devolution of power to citizens as users of services and members of communities - which contributed to the subsequent disregard by the Thatcher government for its recommendations, and the rapid displacement of a primary community social work ethic by one of statutory duty.

Many examples of community based social work had, however, arisen from this period. These echoed the increasing focus in both central and local government on policy for active citizenship and community renewal. In this context, citizenship and community engagement were pursued with increasing momentum as key principles for public policy, with communities increasingly seen as key to localising forms of democratic accountability and as sites for civic participation (Newlove 2011). Local Strategic Partnerships and Area Agreements became the key strategic means of advancing ideas of boundary–spanning active citizenship and social solidarity in practice from 2000 onwards (Geddes et al. 2007). The Labour Government’s vision for strong and prosperous communities further shaped and promoted these goals in its White Paper of 2006 (Department for Communities and Local Government 2006). More recently, the 2018 Civil Society Strategy articulated a vision for creating social value from civil society: communities thriving through partnership and reciprocal contribution (Her Majesty’s Government 2018a).

Undoubtedly this policy trajectory is, in part, determined less by a moral case for local empowerment than by a politically contested imperative for social cohesion (as set out in the Integrated Community Strategies Action Plan for building strong, integrated communities (Her Majesty’s Government 2019)), and by a policy drive that seeks to legitimate the displacement to communities of responsibility for aspects of the public service role which, from a decade of financial ‘austerity’, public services can no longer provide. Nonetheless, it is a trajectory from which multiple pointers to the value of local citizenship perspectives could be drawn, to inform a paradigm for integration; one in which meeting the claim of the individual citizen for ‘personalised’ services over which they exercise individual control is seen not as separate from, but as integral to building the social or community capital on which they depend - and to which as citizens, they contribute.

A paradigm for this kind of integration does, though, appear elusive. Public Health strategy routinely centralises the value of population level initiatives aimed at community wellbeing impact (Public Health England 2015) and within this, the ‘asset-based approach’ features consistently as an expression of the community’s potential contribution in promoting individual health and preventing illness. However, the discourse of integration fails more generally to stimulate a practice in which the value of these different policy traditions is realised, leaving individual and community dimensions of health in largely separate domains. This matters, not least because of the current policy priority of addressing loneliness and social isolation (Her Majesty’s Government 2018b).

## Social Prescribing

The current policy imperative of social prescribing crystallises the need for action in this area. It also highlights a fresh challenge for health and social care practitioners in bridging the domains of public services and local communities to support people with a range of health problems to access opportunities for participation in these communities. Requiring a step-wise implementation centred on primary care networks, social prescribing policy intends to stimulate innovation and systematise practices to which primary care practitioners have, in some measure, long been committed. In articulating intended outcomes for ‘communities, the service system and people’ and in the new provision of a thousand link workers and a dedicated Social Prescribing Academy to achieve them (NHS England 2019b), it appears possible in principle that a strongly integrative approach to both individual and community domains could now move centre stage.

The evidence base for social prescribing, however, is less well developed than UK policy documents may imply. The most rigorous systematic review of social prescribing (Bickerdike et al. 2017) included only 15 studies, of which only one was a randomised controlled trial, and this conducted over 20 years ago (Grant et al. 2000). This review found the evidence to be of low quality, though most studies were positive about social prescribing. Later descriptive reviews have reached similar conclusions (Chatterjee et al. 2018; Pescheny et al. 2018). A further review which explored the process of social prescribing synthesised findings from 109 studies in four categories: exercise (*n* = 66), green prescriptions (*n* = 7), arts on prescription (*n* = 5) and generic social prescribing schemes (*n* = 32) (Husk et al. 2020). This review highlighted the importance of context and capacity, but also that social prescribing should be developed in line with complex intervention and behaviour change approaches. It is recognised that further high quality research is required (Public Health England 2015; National Institute for Health and Care Excellence 2016), but approaches that shift the focus from individual to community wellbeing must be informed by relevant theory.

It is likely that a shift in practice to focus on community wellbeing may take some time. Sitting within the broader policy of universal personalised care (NHS England 2019c), and taking its place as a component in an ‘all age whole population approach to Personalised care’ model, it can be argued that the genesis for social prescribing owes significantly more to the individualisation of social diagnosis than to the collective development of social solutions. This is not to contest the importance of personalisation as a way to understand the necessarily individual and often complex nature of need, nor for meeting it with the sensitivity and optimism that, in relation to mental health, is the ethical heart of the recovery approach (National Voices and Think Local Act Personal 2014). But while the precepts of personalised health care are largely unarguable, its core application as a tool for personal independence can be seen as a counterweight to the cause of encouraging *inter*dependence, or worse, through its association with individual budgets, in the particular context of a decade long politics of harmful public service cuts (Power 2014), as having provided for an unjust shift of responsibility for these cuts from the public to the individual domain (Spicker 2013).

Equally, the impacts of these cuts are likely to be experienced by service providers as having compressed service access to those who fall within increasingly narrow eligibility thresholds. Even less time is available for the involvement of their staff in a whole systems approach to the learning that necessary shifts in professional culture demand.

The challenge to professional engagement in a practice with, and for communities often rests on the truth that the meaning of ‘community’ is theoretically complex and impracticable. It is a challenge that needs to be taken seriously. Clearly, invoking ‘community’ as a single definitional category is to deny the complexity and diversity of communities in a way that makes no sense, and a linear vision of what constitutes community will afford little progress in this area. Yet neither is complexity in itself a justification for partitioning off from the professional sphere the potential to understand and mobilise the social context of people’s lives, especially in the high profile context of injunctions to address loneliness and isolation (Her Majesty’s Government 2018b). Appreciation of an individual’s identity, or more accurately their multiple identities, needs to take account of the part that ‘community’ plays in this. The importance of practitioners having a conscious ‘literacy of community’ is thus paramount.

## A Literacy of Community

Multiple identities imply membership of multiple communities. Communities of neighbourhood, interest, friendship, employment, faith or politics may, with many others, all play a part in the formation, authentication or expression of individual identity. While both health and social care professionals may extrapolate communities of significance to individual patients or clients, this is rarely the starting point that it needs to be if a holistic approach to care or support is to be offered. Moreover, viewing the needs and potential of an individual as a whole person implies an understanding of the meaning which an individual will attach to their whole social system; the value and significance that they associate with its key social components. Clearly the networks to which individuals relate will feature in their view of treatment and support possibilities and a careful appreciation of these should be a central starting point for professional engagement, particularly where the basis for engagement is currently concerned principally with the diagnosis of need and the provision by which it might be met. With an understanding or ‘literacy’ of what community means to individuals comes both the scope to tailor the service response in a personalised way and to understand the potential value of those communities of significance as partners in care or support and as legitimate settings for the exercise of an individual’s civic participation. In knowing the importance of asking ‘what does community mean to this person in front of me?’, the worker is likely to be better equipped to plot a course through complexity and identify what their own part might be in enabling community connection.

In the context of social prescribing it will, in summary, be important for service agencies to recognise and enable the role of the professional as catalyst in helping to build community, as well as individual, capacity. It will also be important for them to formulate a bridge between the two, investing in this form of engagement for the social return that we know the deliberative engagement of communities as social network assets can represent.

## From Individual to Community Wellbeing

Social prescribing typically addresses individual-level outcomes such as social isolation, loneliness, or a lack of individual connection to local resources. Loneliness, in particular is of significant concern in the UK and Government strategies have been developed to address it (Her Majesty’s Government 2018b; Welsh Government 2019). It is apparent across the life course (Victor and Yang 2012) and is associated with depression (Erzen and Çikrikci 2018), increased difficulties in activities of daily living (Shankar et al. 2017), increased health service usage (Gerst-Emerson and Jayawardhana 2015) and increased mortality (Holt-Lunstad et al. 2015).

As a predominantly subjective experience, loneliness is conceptually distinct from the more objective concept of social isolation, which denotes an absence of contact with people (Zavaleta et al. 2017). Although loneliness can occur in crowds, social isolation is often an antecedent of loneliness. It is therefore important to support people to maintain or make new social connections. However, as loneliness refers to individual perceptions of self and one’s social environment, it is more closely aligned to an individual model of health. Loneliness, as a characteristic of individuals, has therefore become a target for social prescription.

This focus on individuals however, neglects the social environment in which people live. As evidence suggests that community connections are associated with lower levels of loneliness, particularly in deprived communities (Kearns et al. 2015), it is important to support the development of opportunity for social connection at both individual and community levels. Higher degrees of community engagement are associated with lower degrees of social isolation (De Koning et al. 2017). The interconnectedness of resources, groups as well as individuals in any given community can serve to promote social contact and prevent isolation and loneliness, thereby improving individual health outcomes.

Community-level outcomes are not currently foregrounded in the role of a social prescribing link worker as they work with individuals. However, community enhanced social prescribing (CESP) recognises that individuals enrich the civic health of communities by developing opportunities for more active engagement with them. Communities are thus not only potential sources of health benefits for individuals, but they provide opportunities for people to enrich existing capacity *and* develop new assets for the benefit of all. By integrating a deliberative community development dimension into social prescribing, CESP works towards enhancing community as well as individual wellbeing. It helps to strengthen the fabric of civic participation and develop community citizenship, all core components of a healthy community (Holden 2018).

The CESP process should result in an increase in ‘sense of community’ (McMillan and Chavis 1986) for both individuals and communities. This is an important outcome for social prescribing in general, and the CESP model in particular, as it supports people to enhance their connections with, and contributions to their communities, as well as deriving benefits from these. The Brief Sense of Community Scale (Peterson et al. 2008) is one approach to measuring this as it measures needs fulfilment, community membership, community influence and emotional connection to community and has been widely used internationally (Wu and Chow 2013; Wombacher et al. 2010; Coulombe and Krzesni 2019; O’Connor 2013).

CESP is conceived as a way to connect professional practice in primary care networks with communities. It aims to impact positively on the culture of primary care practice and provide a way to connect it with community assets, whilst recognising that communities are dynamic and that capacity-building may be required. CESP brings a focus on community wellbeing into social prescribing. To arrive at this, we have integrated two existing models and bodies of evidence: that of Connected Communities and Connecting People.

## Connected Communities

Connected Communities (CC) methodology is founded on the activity and experience of a three year Big Lottery funded study conducted by the RSA in partnership with the University of Central Lancashire (UCLan) and the Personal Social Services Research Unit (PSSRU) at LSE in seven sites across the UK (Parsfield et al. 2015) Incorporating key ideas concerning the co-productive engagement of communities in mental health inclusion, (Morris and Gilchrist 2011; Brophy and Morris 2014; Morris 2012), the study blended deliberative community engagement with social network analysis in a staged process aiming to understand the nature, value and potential of social networks for wellbeing at a local community level and to enable local communities then to apply this in developing, implementing and evaluating an intervention.

Firstly, a local community partnership involving the voluntary sector, community and civil society sector organisations is identified to work with the research team. Through this community partnership, participatory community research is conducted on the basis of an identified issue, set of issues or challenges. These are diverse and in our work to date have included the isolation of single mothers, the exclusion of long term mental health service users, the fragmentation of communities and the lack of social cohesion, the engagement of young people in communities through social media and the integration of the life of an educational academy into that of the community of which it is part.

Community members from the study area become researchers in their own community, receiving training and support from the university. Community researchers administer a community survey, collecting data on people’s experience of local connections measured by the links and contacts that enable people to seek help; that embody important forms of trust and mutuality and that have an impact on wellbeing. These data are then translated into social network maps that depict visually the clusters, type, density and range of individual network relationships within the study area, alongside data on levels of loneliness, mental wellbeing, and residents’ satisfaction with, and sense of community. This information is presented to the community itself through a reflective, focus group based process involving the community partnership, the researchers and respondents. Casting fresh light on who members of the community are to each other, this process can facilitate communities in assessing how connections can be mobilised to improve capacity, and lead to designing bespoke interventions to address the commonly identified issues or development challenges.

Funding for future interventions is secured and on this basis, the intervention is developed and implemented over time by the community partnership (with academic support as required for the technical, analytical and economic aspects of the intervention as it develops). The resulting intervention may be small scale – for example, the establishment of a project that provides social connection and support opportunities for previously isolated single mothers or the agreement of multiple community agencies to synthesise their activities and collaborate in disseminating information to grow previously unseen community connections and assets. A second project involves evaluation of the intervention, which, critically, includes an economic analysis. Evaluation shows that these projects invariably become focal points for a broader approach to sustainable local community activity and additional projects for which there is, by then a sufficiently convincing local community infrastructure to enable successful funding to be sought (Parsfield et al. 2015). In enabling different forms of network potential to be identified and understood, the CC approach has particular relevance to the wellbeing of community members. It offers preventive services a way of strengthening their knowledge base for forms of practical prevention at primary, secondary and tertiary levels based on engaging professional staff jointly – or co-productively with their communities.

## Connecting People

**Fig. 1:**
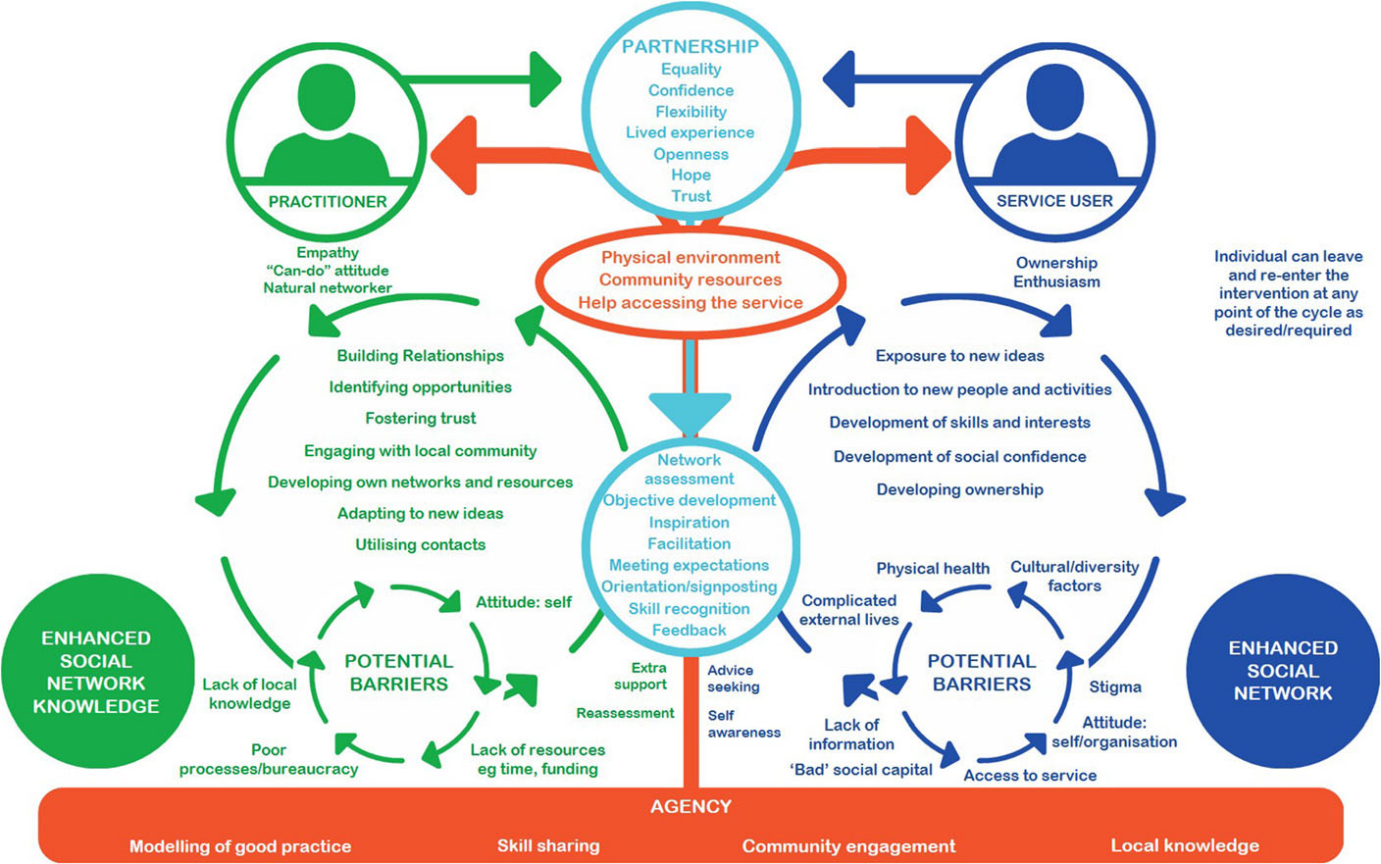
Connecting people model

Connecting People (CP) (Webber et al. 2016) is a dynamic model of practice which aims to enhance an individual’s social network (see Fig. 1). It has been developed from good practice in a range of statutory and voluntary sector agencies in supporting people to make new social connections (Webber et al. 2015).

The relationship of the worker and the individual (a ‘shorthand’ definition for service user, patient or citizen etc) is central to the model, though it is an evolving, mutual relationship which is not typical of traditional ‘clinician-patient’ roles. Conceived as spinning circles, the process requires a partnership where both circles revolve at a pace to suit both the worker and the individual. The circular motion also indicates that the intervention process is a complex, rather than a linear one, as the outcomes do not always emerge predictably as a direct consequence of intervention. Instead, social networks are enhanced as a bi-product of this model. New relationships could form, mutuality be developed and the potential for reciprocity created at any point in the intervention process. The circles are represented as Catherine wheels, with the sparks emitted in all directions representing the unpredictability as to whether or when social networks are enhanced.

The agency in which the intervention occurs – whether this is a statutory service, a voluntary or private sector organisation, a social enterprise, or something else – is crucial. It is depicted on the model as underpinning and being core to the intervention. This demonstrates the responsibility of the agency to support the rest of the process, since without a supportive agency, it is much harder for the rest of the intervention to run smoothly.

The larger circle on the right of Fig. 1 represents the process that an individual undertakes which can lead to social network development. Every instance is different, but in general, the process involves catalysing ideas and experiences. This is where the person is exposed to new ideas and activities, or has their existing ones encouraged and developed. This process may introduce them to new people and activities, further develop their skills and interests and enhance their social confidence. An ultimate goal of this process is to develop networks with new people and organisations which enhance that person’s access to social capital.

The process that the worker follows, (represented by the larger circle on the left of the model), is of equal importance in the intervention process to that followed by the individual. This assumes that the worker will need to develop their own social network knowledge in order to support the individual on their journey. Workers will need to build relationships with the person and often their family, friends and local community, as well as with other local organisations. They will need to foster trust through their reliability and interpersonal skills; identify opportunities; engage with the individual’s local community; develop their own networks and resources and remember these for future use; adapt to new ideas; and utilise their contacts in the process of supporting the person they are working with. It is important that the worker can think creatively and use their resources effectively.

Possible barriers to social network development are represented on the model as two counter-rotating circles which frustrate the motion of the two main circles. Barriers can be diverse and frustrate both the worker or the individual, so potentially posing considerable challenges. The worker and the individual need to work together to overcome the potential barriers to ensure the intervention cycle progresses. Our research (Webber et al. 2019) has found that when these systems and processes occur, and the intervention process moves in the dynamic way that is envisaged in the theoretical model, the outcomes will include an enhancement in the individual’s social network, thereby increasing their access to social capital (Webber and Huxley 2007).

## Community-Enhanced Social Prescribing

CESP is a conceptualisation that utilises the two models as described above to bring together the embedded assets, networks and resources of local communities in order to support individuals who are seeking to improve their wellbeing. It requires a coordinated approach from local agencies which looks beyond the needs of individual organisations, to building environments that help people to help themselves. This approach helps isolated people to engage with local networks, resources and community assets; a shift towards a focus on the enabling environment of the kind indicated in the NHS Long-Term Plan (NHS England 2019a).

One essential component of an enabling environment is that of repeated opportunities for multi-directional collaborations for health and care. Over time, the co-creativity that emerges from such activity builds networks of high-performing teams and local communities for health. Geographic areas provide opportunities for such shared development and we envisage CESP as working within primary care networks, which cover populations of between 30,000 and 50,000 people. To realise this ambition at scale, the whole system needs to support such localism through processes that have been described by Thomas as ‘community-oriented integrated care’ (Thomas 2017).

Change is required at two levels to create the conditions in which CESP can operate. Firstly, at the organisational and systems level within the primary care network, work needs to be undertaken to align organisational objectives with a shared focus on community wellbeing. This could involve a variety of methods, including whole system events using the large group method of real-time strategic change (Jacobs 1997); experienced-based co-design for stakeholders to reflect on data in the light of their experiences and participate in coordinated improvements; or using learning sets for locality leadership teams, local organisations and citizens to consider how best to make CESP work for them.

To inform the process of organisational change, we propose convening a local community citizens’ panel of six to twelve volunteers reflecting the socio-economic and cultural geographies of the local area. These volunteers will be members of the public who are active within the organisations, networks and businesses that are embedded within these communities and have a strong local knowledge. They will be appointed for a 12 month period after which time the panel would be refreshed. They would play a key role in mapping community assets (see below); steering the social prescribing initiative and the attached link worker; providing a strategic community alliance for the primary care network thus helping to shape its approach to community engagement. The citizens’ panel would also have the potential to participate in the governance of the primary care network.

Secondly, at individual level, a social prescribing referral system for agreed target groups (e.g. people with long-term conditions or mental health problems) will need to be established. Link workers would be trained in the Connecting People approach so that they can use it with the people with whom they work. This approach will enable CESP to be applied in locally-relevant ways that also help to incrementally transform the whole system towards effective use of local networks, resources and community assets. There are two essential processes of CESP which can be summarised as ‘contextualising the community’ and ‘engaging with the community’.

## Contextualising the Community

The primary care network facilitates the citizens’ panel to map local assets, networks and resources, which are accessible to members of the local community, particularly those which are informal or not widely publicised. It is important that this is not merely a list of voluntary organisations in the local area, but also includes knowledge of the local neighbourhood networks which may be more informal and known only to local panel members. If the primary care network wished to fund it, panel members could become community researchers and, as in the Connected Communities model described above, collect data on social connectivity and asset utilisation within the local population. In any event, this process must be iterative and continuous since it needs to reflect the individual, diverse and dynamic characteristics of active communities, and use this to inform the organisations that are of key importance to effective social prescribing, particularly primary care networks.

Using appropriate physical or social media, this multi-dimensional resource is then shared in public spaces within the local community, such as primary care surgeries, public noticeboards, community centres and social media groups, with members of the public invited to contribute and shape it further. Contact details for people associated with the assets are also collated so that they can be accessed as required. The citizens’ panel is responsible for ensuring that these maps are reviewed and revised regularly.

## Engaging with the Community

Each primary care network employs a link worker who is responsible for social prescribing. The link worker works closely with the citizens’ panel to fully understand the assets of the local community, and also supports it and feeds into the mapping process. They utilise a model of social prescribing informed by Connecting People, which requires full and active engagement with the community with whom they are working. Link workers engage with people within primary care settings who are seeking to improve their wellbeing by engaging with local groups, networks, resources, activities or assets. They follow the Connecting People steps of establishing readiness; mapping the individuals’ existing networks and access to local community assets; setting goals for enhancing their wellbeing and planning with which local resources might assist; supporting them to engage with community resources; reviewing with them their progress towards their goals and supporting them to overcome barriers to community engagement. Link workers’ engagement with local people, the citizens’ panel and the wider primary care network gives them an important role in ensuring that the local asset map is a dynamic resource that is kept up to date, is relevant and fit for purpose. As well as using it in their daily work, they will also continually update it and promote its use in the wider community. Working with the citizens’ panel, they will also help to identify gaps in local provision. It is hypothesised that increased knowledge and use of local assets, resources and networks will bring benefits for both individuals and communities.

## Theory of Change

**Fig. 2:**
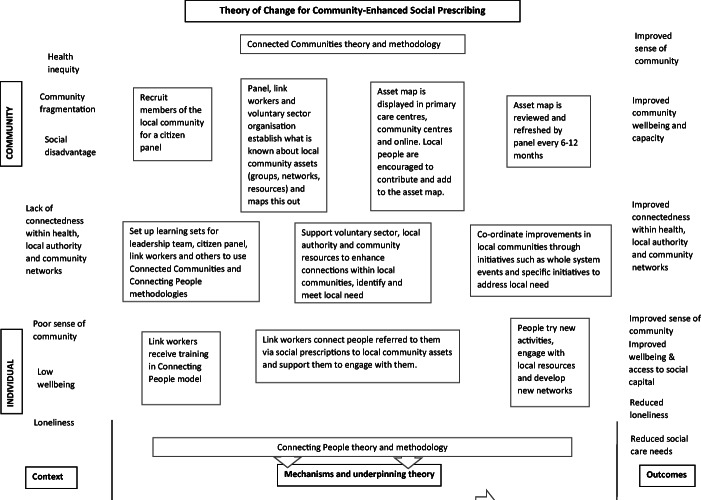
Community enhanced social prescribing theory of change

It could reasonably be expected that operationalising the CESP model in the way described here will achieve a number of key outcomes as suggested in the theory of change model (Fig. 2).

Figure 2 summarises the processes described above. It assumes a context characterised by social and health inequalities, community fragmentation and social disadvantage, where local services are not well connected and individuals experience loneliness, low wellbeing and a poor sense of community (Fig. 2, left column). Social prescribing link workers use the Connecting People model to support individuals to engage with local community assets (Fig. 2, bottom of middle column). The link workers draw upon the local asset map developed by the citizens’ panel, which is informed by Connected Communities methodology (Fig. 2, top of middle column). The third set of processes in the theory of change model relate to organisational change whereby connections between organisations are enhanced, awareness of community assets is increased and whole system events bring people and organisations together to consider how best to meet emerging local need (Fig. 2, middle centre of middle column).

If fully operationalised, CESP should enhance individual and community outcomes (Fig. 2, right column). For individuals able to utilise and contribute to community assets, we would expect them to experience improvements in their sense of community, general wellbeing and access to social capital. Greater community engagement could also contribute to reducing loneliness, social care needs or help to support the management of long-term conditions. These outcomes are expected on the basis of findings of Connected Communities, Connecting People or similar initiatives to improve community engagement (e.g. Parsfield et al. 2015; Webber et al. 2019; O'Connor 2013).

Uniquely for a social prescribing model, we anticipate that CESP will improve community-level outcomes (Fig. 2, right column), suggesting that the model has the potential to benefit all community members irrespective of their receipt of a social prescription. While outcomes on this scale may take longer to become apparent, we anticipate that they will give rise to improvements in a collective sense of community, community wellbeing and the potential for community capacity to occur in the future. Greater awareness of a community’s assets should lead to an increased use of community resources and local investment in them. The citizens’ panel will also help to identify gaps which, if addressed, will further enhance community wellbeing, thus creating a virtuous cycle.

Improved outcomes are dependent on changes in the local health and social care infrastructure. Local primary and secondary health care services need to work towards being part of a whole system, integrated with local community and voluntary organisations (Fig. 2, middle of central column). This can be challenging where there are significant differentials in power, security of funding and professional priorities between agencies. However, where inter-agency coherence can be achieved, the improved connectedness can stimulate local initiatives which can improve community wellbeing in ways not previously foreseen, such as those concerned with improved resilience or crisis response.

## Discussion

Community wellbeing is a multidimensional construct encompassing a number of domains (Phillips and Wong 2016; Sung and Phillips 2018). There are many influences on the subjective and objective wellbeing of a community. It is unlikely that a single factor has an over-riding influence, but inter-connections between domains are likely to be important. Uniquely for social prescribing, the CESP model described in this paper is one that integrates individual and community level activity and, uniquely for social prescribing, produces outcomes for both individuals and communities. It foregrounds the role of connectedness within local community and health networks and is an integrated contextual intervention to optimise outcomes at individual and community levels. Enhanced community engagement is likely to reduce the loneliness and social isolation of individuals, maximise their reciprocal contribution to their community and improve the availability of local resources for the wellbeing of the community as a whole.

It remains to be seen if the implementation of CESP can address health inequalities within and between communities. This is a particularly pressing problem in England where life expectancy has fallen in the last ten years in the most deprived communities outside London (Marmot et al. 2020). The social determinants of health are not being adequately addressed by public policy, health and social care services or the voluntary and community sector. This is partly as a result of a decade of austerity in which services have been starved of funds and investment, but it is also due to the ways in which individualism, appearing as an implicit guiding principle and strongly politicised priority for society, has worked to de-emphasise collective action and its value. Individualism and collectivism have opposite associations with loneliness (Heu et al. 2019), and a shift towards policies which promote collective action may also improve individual outcomes.

At the time of writing in 2020 and in light of the Covid-19 pandemic, communities are finding new ways of coming together to identify and support each other, in particular, people who are vulnerable and therefore self-isolating. Mutual aid groups have been set up in neighbourhoods; neighbours are establishing WhatsApp groups in their streets; communities are looking out for those without friends or family who may need practical help with shopping or medicine collection and befriending groups are being set up offering phone or online contact. We are witnessing community organising on an unprecedented scale; collective action to meet individual need. Like the future impact of the Covid-19 crisis itself, the extent to which community groups will work alongside statutory health and social care services, in supporting the fabric of communities is unknown. However, it is apparent that when communities self-organise in the way proposed by the CESP model, there is strong potential for improved individual and collective outcomes.

## Conclusion

The CESP model brings together two established approaches, Connected Communities and Connecting People, with their own different and distinctive evidence bases, to form an integrated whole through which we can address the twin dimensions of working with individuals and connecting them to communities empowered to better understand their assets and needs. Both are required in an integrated model for the success of social prescribing. However, this is as yet unproven. Research is required in different contexts to evaluate how contextually-bound the model is likely to be, and which conditions need to be present for community wellbeing to be enhanced. Civic participation as a necessary dimension of being a citizen is envisaged as a necessary precondition for the success of the model. We suggest that community wellbeing will be enhanced by improved civic participation through a reciprocal wellbeing transfer among individuals and to the community as a whole, uniquely benefitting both individuals and communities.

## Data Availability

Not applicable.
